# Feature optimization based on improved novel global harmony search algorithm for motor imagery electroencephalogram classification

**DOI:** 10.3389/fncom.2022.1004301

**Published:** 2022-12-16

**Authors:** Bin Shi, Xiaokai Chen, Zan Yue, Feixiang Zeng, Shuai Yin, Benguo Wang, Jing Wang

**Affiliations:** ^1^Xi’an Research Institute of High-Technology, Xi’an, Shaanxi, China; ^2^Rehabilitation Medical Center, Huizhou Third People’s Hospital, Huizhou, China; ^3^Institute of Robotics and Intelligent System, School of Mechanical Engineering, Xi’an Jiaotong University, Xi’an, China; ^4^iHarbour Academy of Frontier Equipment (iAFE), Xi’an, China; ^5^Department of Rehabilitation Medicine, Longgang District People’s Hospital of Shenzhen, Shenzhen, China; ^6^Department of Rehabilitation Medicine, The Second Affiliated Hospital of The Chinese University of Hong Kong, Shenzhen, China

**Keywords:** brain-computer interface (BCI), common spatial pattern (CSP), frequency band, time interval, improved novel global harmony search (INGHS), electroencephalogram (EEG)

## Abstract

**Background:**

Effectively decoding electroencephalogram (EEG) pattern for specific mental tasks is a crucial topic in the development of brain-computer interface (BCI). Extracting common spatial pattern (CSP) features from motor imagery EEG signals is often highly dependent on the selection of frequency band and time interval. Therefore, optimizing frequency band and time interval would contribute to effective feature extraction and accurate EEG decoding.

**Objective:**

This study proposes an approach based on an improved novel global harmony search (INGHS) to optimize frequency-time parameters for effective CSP feature extraction.

**Methods:**

The INGHS algorithm is applied to find the optimal frequency band and temporal interval. The linear discriminant analysis and support vector machine are used for EEG pattern decoding. Extensive experimental studies are conducted on three EEG datasets to assess the effectiveness of our proposed method.

**Results:**

The average test accuracy obtained by the time-frequency parameters selected by the proposed INGHS method is slightly better than artificial bee colony (ABC) and particle swarm optimization (PSO) algorithms. Furthermore, the INGHS algorithm is superior to PSO and ABC in running time.

**Conclusion:**

These superior experimental results demonstrate that the optimal frequency band and time interval selected by the INGHS algorithm could significantly improve the decoding accuracy compared with the traditional CSP method. This method has a potential to improve the performance of MI-based BCI systems.

## Introduction

A brain-computer interface (BCI) system is utilized to sense and transform the electroencephalogram (EEG) signal from the scalp into commands to control external devices and help users to accomplish tasks (Wolpaw, 2002; Cervera et al., 2018; Lazarou et al., 2018; Xu et al., 2018, 2021; Mudgal et al., 2020; Rashid et al., 2020). The EEG is commonly used for brain analysis (Nicolas-Alonso and Gomez-Gil, 2012). A large number of researchers pay more attention to the research of BCI based on motor imagery (MI). The mechanism of EEG-based MI-BCI is that the subject can autonomously regulate the sensorimotor rhythm (SMR) through performing the MI task (Pfurtscheller et al., 1993, 2006). The SMR is characterized by power changes in specific frequency bands (8–30) over the sensorimotor cortex. The modulation of SMR generates contralateral preponderant event-related desynchronization (ERD) and synchronization (ERS), which are short lasting attenuation and enhancements of SMR. It is generally accepted that ERD/ERS happens in the different spatial-frequency-temporal domains when different subjects execute MI task, causing difficulty in extracting effective features (Hamedi et al., 2016; Li et al., 2019; Jiao et al., 2020).

A standard BCI system comprises a signal acquisition unit, signal processing unit, controlling unit, and application or feedback unit. The signal processing unit further includes three parts, namely, preprocessing, feature extraction, and feature classification. The effective feature extraction method is very important for the recognition of MI intention (Rasheed, 2021). Various feature extraction techniques are used for the feature extraction of EEG-based MI, such as Principal Component Analysis (PCA) (Mirzaei and Ghasemi, 2021), Wavelet Transform (WT) (Sreeja et al., 2017), Fast Fourier Transform (FFT) (Chaudhary et al., 2019), and Common Spatial Pattern (CSP) (Miao et al., 2017b). Currently, the CSP is one of the most popular feature extraction methods which can effectively extract the spatial information of ERD/ERS (Siuly and Li, 2015). However, due to the influence of nonstationary in EEG and inherent defects of the CSP objective function, the spatial filters, and their corresponding features are not necessarily optimal in the feature space used within CSP. On the one hand, internal feature selection method of CSP based on L1-norm and Dempster–Shafer theory was proposed to result in a significant increase in the performance of MI-based BCI systems (Jin et al., 2021). On the other hand, the selection of frequency band and time interval has a great influence on the CSP features. Under the same experimental paradigm, the most reactive frequency band and response time interval of different subjects performing the MI are distinct (Ramoser and Muller-Gerking, 2000). It is demonstrated that the classification performance of the BCI system could be enhanced through the selection of the distinguishable frequency band, maximum discriminative time interval, and high-separability power channels for specific participants (Ince et al., 2009).

The main study for the select of frequency band and temporal interval focuses on the following aspects. (1) Frequency band optimizing: the sub-band common spatial pattern (SBCSP) was reported (Quadrianto et al., 2007). Mutual information-based feature selection method was employed to select distinguishable pairs of frequency bands in filter bank common spatial pattern (FBCSP) algorithm that yields superior classification performance compared with CSP and SBCSP (Kai et al., 2008). Discriminative filter bank common spatial pattern (DFBCSP) was reported to extract the optimal frequency band by means of fisher ratio and achieved better classification accuracy (Thomas et al., 2009). A sparse filter band common spatial pattern (SFBCSP) was introduced to optimize the frequency domain (Zhang et al., 2015). (2) Temporal domain optimizing: the novel correlation-based time window selection (CTWS) algorithm was applied for MI-based BCIs, and the results indicate that compared to the classical CSP method, the CTWS algorithm significantly enhanced the average classification accuracy of healthy participants and stroke survivors (Feng et al., 2018). (3) Frequency-temporal optimizing: the frequency-time synthesis optimizing method for the MI-based BCI system was reported to adapt to the individual difference (Tao et al., 2004). The local discriminant bases algorithm was proposed to find the starting time of the ERD/ERS in the sub-band of the EEG (Ince et al., 2007). Fisher discriminant analysis-type *F*-score approach was developed to simultaneously optimize the frequency-time domain for multi-class classification (Yang et al., 2017).

All these mentioned works have demonstrated that optimizing frequency band or time interval could contribute to yield better classification results. However, most of the studies aim to find the optimal time-frequency parameters in multiple sub-bands and time intervals based on the same bandwidth and time window length. The fixed bandwidth and time window length is not individual variability. Furthermore, although most of the proposed algorithms can automatically optimize the frequency band and time interval, they are independent of each other in the selection process. Since the optimal CSP features are determined by the mutual influence of both frequency and time parameters, the above sequential select procedure method is not the optimal solution in terms of finding the optimal frequency band and time interval. In essence, it might be the best choice to select simultaneously frequency-temporal parameters in the optimization process so that the CSP features obtained by the optimal frequency-time parameters can enhance the classification performance for MI-based BCI systems. Recently, some meta-heuristic algorithms were introduced to optimize frequency-temporal parameters. The particle swarm optimization (PSO) algorithm was utilized to select the optimal frequency and time parameters to extract the effective CSP features (Xu et al., 2014). The artificial bee colony (ABC) algorithm was proposed to solve the frequency-temporal optimization problem (Miao et al., 2017a). However, most of these algorithms require complex operations when creating an offspring. Moreover, these algorithms have many options for parameters and need a relatively long run time to find the global optimal solution.

Harmony search (HS) algorithm was firstly proposed in 2001 (Geem et al., 2001). Since then, HS and its variants have been reported and widely applied to various optimization problems (Mahdavi et al., 2007; Omran and Mahdavi, 2008; Zou et al., 2010). An improved novel global harmony search algorithm (INGHS) was proposed (Ouyang et al., 2015) and the results indicate that the INGHS algorithm performs better than PSO and ABC algorithms in solving the reliability optimization problem. INGHS algorithm has been successfully applied to data clustering and engineering design optimization problems (Ouyang et al., 2018; Talaei et al., 2020). To sum up, the iterative updating principle of INGHS is simpler than PSO and ABC, with faster convergence and better performance. The PSO and ABC algorithms can find good time-frequency parameters in the application of BCI system, but the time cost is high. Therefore, in our work, the INGHS algorithm is introduced in this article to solve the combined frequency-time optimization problem for more accurate MI-related EEG classification. Extracting CSP features from MI EEG signals is often highly dependent on the selection of frequency band and time interval. The CSP features obtained with fixed frequency band and time interval might affect the classification performance of MI-based BCI systems. To address the above drawbacks, the contribution of this work is:

1. Propose an approach based on an INGHS to optimize frequency-time parameters for effective CSP feature extraction.
2. Conduct a set of experiments validating the effectiveness of the proposed method.
3. Compared with PSO and ABC, INGHS algorithm can converge to the global optimal solution faster, so it is helpful for specific subjects to find the optimal time interval and frequency band in the actual offline experiment in a shorter time, thus shortening the offline calibration time.

Therefore, the rest of the article is organized as follows. The applied datasets and methods are described in section “Methods and materials.” Then, in the section “Results and discussion,” we describe the results of channel selection, test classification comparison, analysis of frequency-temporal parameters optimization, and computational time comparison. Finally, this study is summarized in section “Conclusion.”

## Methods and materials

### Electroencephalogram data description

**FIGURE 1:**
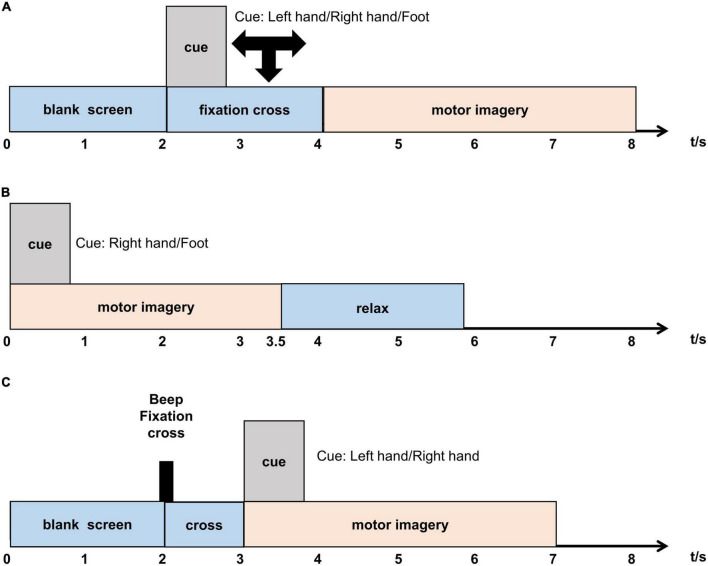
**(A–C)** Timeline of a trial of three datasets.

(1) Data 1: The first dataset was from the BCI Competition IV dataset 1. The EEG signals of seven subjects (“a” to “g”) at 59 EEG electrodes were recorded. The calibration data consisting of 200 trials for each subject was utilized in this study. In each trial, cue show a duration of 4 s, during which each subject performed the corresponding MI (right hand and left hand or foot) tasks. The original data are downsampled to 100 Hz. The timeline of a trial is illustrated in Figure 1A. More details can be found in the following website: http://www.bbci.de/competition/iv/.

(2) Data 2: We used the BCI Competition III dataset IVa for the experimental study. Five healthy participants (“aa” to “ay”) from 118 EEG electrodes were recorded in this dataset. The data are downsampled to 100 Hz. In each trial, cues show a duration of 3.5 s, during which each subject performed the corresponding MI (right hand and right foot) tasks. The timeline of a trial is shown in Figure 1B. More details can be found in the following website: http://www.bbci.de/competition/iii/.
(3) Data 3: The third dataset was from the BCI Competition III dataset IIIa. The EEG signals of three subjects were recorded in this dataset at 64 electrodes but the competition received data of only 60 electrodes. Only the EEG data of left-hand and right-hand are employed in this study due to the binary classification. During each run, the first 2 s were quiet and a cross was displayed at *t* = 2 s. Then from *t* = 3–7 s, the subject executed the imagery task. The sampling rate is 250 Hz and have different numbers of trials for each subject in this study. The subjects are “k3b” (90), “k6b” (60), and “l1b” (60). The timeline of a trial is illustrated in Figure 1C. More details can be found in the following website: http://www.bbci.de/competition/iii/.

### Data preprocessing and channel selection

At first, the continuous EEG data from three datasets are divided into single-trial data and then common average reference (CAR) is applied for the spatial filter to enhance the signal-to-noise ratio (Mcfarland et al., 1997). Moreover, the EEG data are filtered by using a fifth-order Butterworth band-pass filter from 5 to 40 Hz (Miao et al., 2017a).

The channel selection method could not only remove the irrelevant and redundant channels but also reduce the calculation cost for the subsequent time-frequency parameter optimization to obtain better classification performance (He et al., 2013). The discriminative power of each channel is calculated by Fisher’s discriminative criteria (FDC) value between the two classes. First of all, time segmentation is conducted by using rectangular time windows (100 points) and the length of signal (250 points) for datasets 1 (100 Hz × 4 s) and dataset IVa (100 Hz × 3.5 s), and dataset IIIa (250 Hz × 4 s), respectively. The 50% overlapping is used in neighboring t-segments for three datasets. For single-channel, P_ch,*t*_ = log(*var*(x_ch,t_)) is calculated as the feature of each segment, where x_ch,t_ is signal data of *t*-segment of channel *ch*, and P_ch,t_ denotes log-power. Then, the FDC value between two classes is $\phi_{\mathrm{ch},t}={\left({\text{m}}_{1}-{\text{m}}_{2}\right)}^{2}/\left(v\,a\,r\,\left({\text{P}}_{\mathrm{ch},t}^{1}\right)+v\,a\,r\,\left({\text{P}}_{\mathrm{ch},t}^{2}\right)\right)$, where m_1_ and m_2_ are means of P_ch,t_ of all trials in two classes. ${\text{P}}_{\mathrm{ch},t}^{1}$ and ${\text{P}}_{\mathrm{ch},t}^{2}$ denote log-power of two classes, respectively. Finally, the maximum FDC of all *t*-segments is taken as the FDC value of each channel. The FDC values of all channels are arranged in descending order. In the set of FDC values, the first *K* corresponding channels are taken as the optimal channels in this study. *K* denotes the number of the selected channels.

#### Feature extraction and classification

The CSP is a feature extraction method that projects multichannel EEG signals from the two classes into a subspace and decomposes them into different spatial patterns (He et al., 2010; Alvarez-Meza et al., 2015; Nicolas-Alonso et al., 2015; Wang et al., 2020; Mladenović et al., 2022). The CSP algorithm maximizes the difference between classes by simultaneously diagonalizing the covariance matrix and is described as follows:

The *e*-th MI EEG data could be represented as Xe=[x1(t),x2(t),…,xn(t)]Tt=t,0…,T, where *n* is the number of electrodes. X_d_, d ∈ {1,2} denotes the EEG data of class 1 or class 2. The normalized average covariance matrix of class 1 and class 2 are calculated as:


(1) $${\bar{C}}_{\text{d}}=\frac{1}{\text{N}}\,\sum \frac{\left({\text{X}}_{\text{d}}\,{\text{X}}_{\text{d}}^{T}\right)}{\text{trace}\,\left({\text{X}}_{\text{d}}\,{\text{X}}_{\text{d}}^{T}\right)}$$

where *N* is the number of trials for EEG data in a class. Then, the covariance space ${\text{C}}_{\text{c}}=\bar{{\text{C}}_{1}}+\bar{{\text{C}}_{2}}$ consists of mean covariance matrices of the two classes. The eigendecomposition of C_c_ can be expressed as ${\text{C}}_{\text{c}}={\text{U}}_{\text{c}}\,\lambda_{\text{c}}\,{\text{U}}_{\text{c}}^{\text{T}}$. *P* is the whitening matrix: $P=\sqrt{\lambda_{\text{c}}^{-1}}\,U_{\text{c}}^{\text{T}}$. Define ${\text{R}}_{1}=\text{P}\,{\bar{\text{C}}}_{1}\,{\text{P}}^{\text{T}}$ and ${\text{R}}_{2}=\text{P}\,{\bar{\text{C}}}_{2}\,{\text{P}}^{\text{T}}$. Then, *R*1 is calculated by R_d_ = *B*λ_d_*B*^T^. B is the orthogonal matrix and λ_d_ is a diagonal matrix. If R_1_ = *B*λ_1_*B*^T^, then R_2_ = *B*λ_2_*B*^T^, and λ_1_ + λ_2_ = I. When the λ_1_ is closer to *I*, the λ_2_ is closer to 0. Thus, the difference between the two classes is the largest. The projection matrix *W* is calculated as: W = B^T^P. The X^e^ is projected onto Z = WX^e^. The number of features is 2*m* and *m* = 1 in this study. The features f_p_, could be calculated as follows:


(2) $${\text{f}}_{\text{p}}=\text{log}\,\left(\frac{\text{Var}\,\left({\text{Z}}_{\text{p}}\right)}{\sum_{\text{i}=1}^{2\,\text{m}}\text{Var}\,\left({\text{Z}}_{\text{i}}\right)}\right),\text{p}=1,\ldots ,2\,\text{m}$$

In this article, linear discriminant analysis (LDA) and a Radial Basis Function (RBF) kernel-based support vector machine (SVM) are used as classification methods (Jin et al., 2019; Jin et al., 2020; Mladenović et al., 2022). The MATLAB Toolbox (LIBSVM) is used in this study for classification (Chang and Lin, 2011).

### Improved novel global harmony search algorithm

**FIGURE 2:**
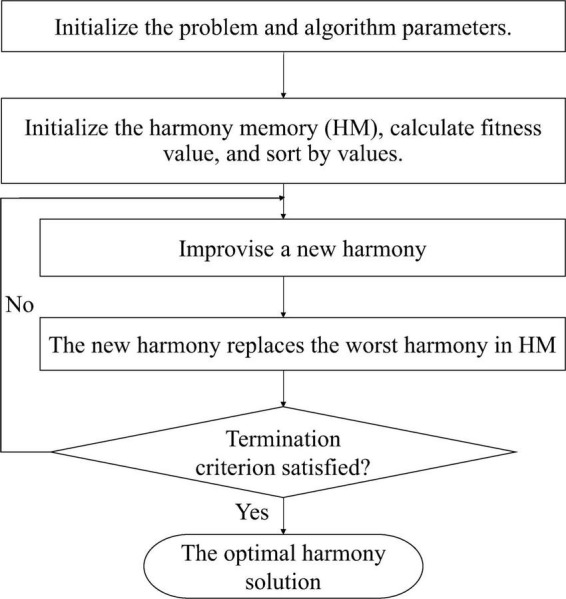
The flow chart of the INGHS algorithm.

Inspired by the music improvisation process, Geem proposed a new meta-heuristic optimization algorithm called harmony search (HS) (Geem et al., 2001). The novel global harmony search algorithm (NGHS) was proposed based on the idea of swarm intelligence of particle swarm (Zou et al., 2010). The NGHS algorithm first initializes the problem and parameters including genetic mutation probability (*P*_*m*_), maximum iteration number, and harmony memory size (HMS). Then position updates and low-probability genetic mutations are used to produce a new harmony. Finally, no matter whether the new harmony is better than the worst harmony in the harmony memory (HM), the new harmony would replace the worst harmony. If the predetermined termination criterion is not met, the above process is repeated. However, the purpose of the position update operation is to move the worst harmony in the HM to the best harmony in each iteration in the NGHS algorithm, which can easily result in premature convergence. Moreover, the algorithm has never considered that other harmony solutions except for the worst harmony can improvise better harmony in each iteration. Therefore, an INGHS algorithm was proposed to boost the quality of the solution and keep the NGHS algorithm from falling into local optimal solution (Ouyang et al., 2015). The INGHS algorithm proposed a coefficient of optimization opportunity, which can dynamically adjust to keep a balance between the exploitation and exploration to enhance the local search ability and accelerate the convergence rate of algorithm. Figure 2 presents the flow chart of the INGHS algorithm.

The INGHS works as follows:

**Step 1:** Initialize the problem and parameters

The optimization problem is defined as minimize (or maximize) *f*(x) such that x_iL_ ≤ x_i_ ≤ x_iU_(i = 1,2, …, n), where the objective function denotes *f*(x), and x is a candidate solution composing of *n* decision variables (x_i_). This step also needs to determine the parameters which include the HMS, genetic mutation probability *P*_*m*_, and the number of iterations (Ni).

**Step 2:** Initialize the harmony memory (HM)

The initial HM is yielded from a uniform distribution in the variable interval [x_iL_ x_iU_], where x_iL_ and x_iU_ are the lower and upper bounds for x_i_, respectively. This is done as follows: ${\text{x}}_{\text{i}}^{\text{j}}={\text{x}}_{\text{iL}}+\text{r}\times \left({\text{x}}_{\text{iU}}-{\text{x}}_{\text{iL}}\right),j=1,2,\ldots ,H\,M\,S$. Where r∼U(0,1), and *f*(x) is the objective function values of each harmony vectors, as shown in Equation 3.


(3) $$\text{HM}=\left[\begin{array}{cccc} x_{1}^{1} & x_{2}^{1} & \cdots & x_{n}^{1}\\ x_{1}^{2} & x_{2}^{2} & \cdots & x_{n}^{2}\\ \vdots & \vdots & \cdots & \vdots \\ x_{1}^{\text{HMS}} & x_{1}^{\text{HMS}} & \cdots & x_{n}^{\text{HMS}} \end{array}|\begin{array}{c} f\,\left({\text{x}}^{1}\right)\\ f\,\left({\text{x}}^{2}\right)\\ \vdots \\ f\,\left({\text{x}}^{\text{HMS}}\right) \end{array}\right]$$

**Step 3:** Improvise a new harmony

Generating a new harmony is called improvisation. $x^{\prime }=\left({\text{x}}_{1}^{\prime },{\text{x}}_{2}^{\prime },\ldots ,{\text{x}}_{\text{n}}^{\prime }\right)$ is the new harmony vector. O(u) is defined as the coefficient of optimization opportunity and its expression is $\text{O}\,\left(\text{u}\right)=1-\sqrt{1-\left(\text{u}/N\,i\right)}$ (*u* is the current iteration). ${\text{x}}_{\text{i}}^{\text{best}}$ represents the *i*-th components of the best harmony (minimum fitness value) in HM, and ${\text{x}}_{\text{i}}^{\text{worst}}$ represents the *i*-th components of the worst harmony (maximum fitness value) in HM. ${\text{x}}_{\text{i}}^{\text{s}}$ is the *i*-th components of stochastic harmony in HM. r is uniformly generated random number in the region of [0 1]. The objective of the new harmony vector f(*x*′) is calculated. The specific procedure is as follows:

For each i ∈ [1, n] do

If r < O(u)


$$x_{R}=2\times x_{i}^{b\,e\,s\,t}-x_{i}^{w\,o\,r\,s\,t}$$

Else


$$x_{R}=\left(1+r\right)\times x_{i}^{b\,e\,s\,t}-\left(1-r\right)\times x_{i}^{s},s\in \left(1,2,\ldots ,\text{HMS}\right)$$

End if

If *x*_*R*_ < *x*_*iL*_


$$x_{R}=x_{i\,L}$$

Else If *x*_*R*_ > *x*_*iU*_


$$x_{R}=x_{i\,U}$$

End if

${\text{x}}_{\text{i}}^{\prime }={\text{x}}_{\text{i}}^{s}+\text{r}\times \left({\text{x}}_{\text{R}}-{\text{x}}_{\text{i}}^{s}\right)$/*position updating*/

If r < P_m_ then

${\text{x}}_{\text{i}}^{\prime }={\text{x}}_{\text{iL}}+\text{r}\times \left({\text{x}}_{\text{iU}}-{\text{x}}_{\text{iL}}\right)$/*genetic mutation*/

End if

End for

**Step 4:** Update HM

If the objective value of the improvised harmony vector *x*′ is better than that of the stochastic selected harmony *x*^s^, we replace the stochastic selected harmony in the HM with *x*′.

**Step 5**: Check the stopping criterion

The iteration is terminated when the maximum Ni is reached. Otherwise, Steps 3 and 4 are repeated.

### An improved novel global harmony search-based frequency-temporal parameter optimization scheme

**FIGURE 3:**
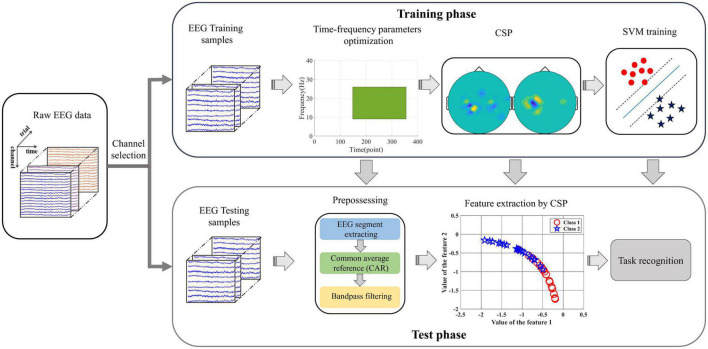
The flow chart of the proposed INGHS-based method.

Firstly, the original EEG data sets were preprocessed by CAR and band-pass filter. Then, the FDC-based method is used for the raw EEG data to select the optimal channel sets. The 10-fold cross-validation is employed to verify the effectiveness of the proposed INGHS method. Specifically, the raw EEG data with channel selection were randomly divided into 10 parts, 9 parts of which were used as training data and the remaining one as test data. Figure 3 presents the flow chart of the INGHS-based frequency-temporal parameter optimization. The proposed method mainly includes training phase and test phase. For the training phase, the INGHS algorithm was used for the training data sets to search the optimal frequency band and time interval. Thereafter, the projection matrix is obtained by CSP algorithm to applied training data extracted with the optimal frequency-temporal parameter. The CSP features is applied to train the SVM model. For test phase, the optimal frequency-temporal parameter is employed for testing samples to extract the EEG segment. Then, the projection matrix is used to extract the CSP features which is putted into SVM model to classification.

It should be noted that the objective function in INGHS algorithm was defined to calculate the fitness value for evaluating the quality of a solution. The objective functions are the mean error rate of fivefold cross-validation.

The INGHS-based approach works as follows:

*(1) Initialize the problem and algorithm parameters.*

The optimization problem is defined as minimize *H* (*f*, *t*) subject to *h*_*iL*_ ≤ *h*_*i*_ ≤ *h*_*iU*_(*i* = 1−4). h_1_ and h_2_ denote the start frequency (f_*start*_) and bandwidth (f_*width*_), while h_3_ and h_4_ denote the starting of time interval (t_*start*_) and the length of time interval (t_*length*_). Therefore, the solution is expressed as {f_*start*_, f_*width*_, t_*start*_, t_*length*_}. In this study, the introduced INGHS algorithm was applied for the training data sets to find the globally optimal solution. The searching ranges for each variable of feasible solution are listed in Table 1. For all subjects, if f_*start*_ + f_*width*_ ≥ 40, then f_*width*_ = 40 − f_*start*_. For datasets 1, if t_*length*_ + t_*start*_ ≥ 4 × 100, then t_*length*_ = 4 × 100−t_*start*_. For dataset IVa, if t_*length*_ + t_*start*_ ≥ 3.5 × 100, then t_*length*_ = 3.5 × 100−t_*start*_. For dataset IIIa, if t_*length*_ + t_*start*_ ≥ 4 × 250, then t_*length*_ = 4 × 250−t_*start*_. The INGHS algorithm parameters (HMS, *P*_*m*_, and Ni) are also determined in this step.

**TABLE 1:** The range of four variables for each feasible solution.

Variable	Datasets		
	IV-1	III-IVa	III-IIIa
*f*_*start*_ (Hz)	5–30	5–30	5–30
*f*_*width*_ (Hz)	5–30	5–30	5–30
*t*_*start*_ (sample points)	1–350	1–300	1–3.5 × 250
*t*_*length*_ (sample points)	100–350	100–300	250–3.5 × 250

*(2) Initialize the HM and calculate the fitness value.*

**FIGURE 4:**
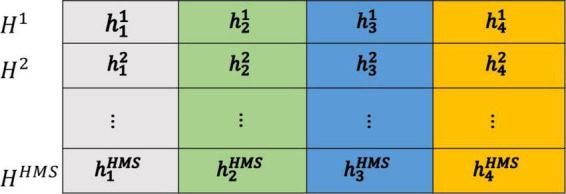
Initialize the harmony memory.

The initial HM is generated from a uniform distribution in the ranges [h_iL_, h_iU_], as shown in Figure 4. Each harmony vector (solution) is applied on the training data. At the same time, the features are extracted by the CSP algorithm based on the training data sets. Frequently, the obtained features is inputted into the LDA (or SVM) classification algorithm to calculate the fitness value H(f, t), and then sort by fitness values.

*(3) Improvise a new harmony.*

According to Step 3 of the INGHS algorithm to improvise a new harmony and calculate the fitness value.

*(4) Update HM and (5) Stopping criterion* are the same as the INGHS algorithm. Finally, the optimal frequency band and time interval are derived. Meanwhile, the test data sets are processed by the optimal frequency band {f_*start*_, f_*start*_ + f_*width*_} and time interval {t_*start*_, t_*start*_ + t_*length*_}. Features are extracted by the CSP filters from the INGHS algorithm. Furthermore, the LDA (or SVM) classifier is utilized to recognize the MI task.

**FIGURE 5:**
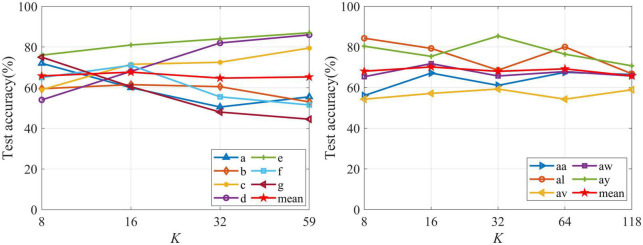
Test classification accuracies of subjects from Data 1 and Data 2 with the change of *K*.

To remove the irrelevant and redundant channels and enhance the classification accuracy and reduce the computational complexity, channel selection methods for MI-based BCI have been widely studied (Miao et al., 2017a; Jin et al., 2019, 2020). Compared with other channel selection methods, the FDC method is widely used and has low complexity, the FDC is selected for channel selection. We investigated the effect of the change of the number of selected channels (*K*) on the test accuracy. The CSP algorithm is used as feature extract method and is not executed frequency-time parameters optimization. The frequency band is 5–40 Hz and time interval is MI time (paradigm setting). The LDA method is utilized to the classification. *K* is tuned from 8 to 59 for Data 1. For Data 2, *K* is tuned from 8 to 118. Figure 5 presents the test classification accuracies of subjects from Data 1 and Data 2 with the change of *K*. The test accuracy obtained by each *K* is the average of 10-fold cross validation. According to the results in Figure 5, the test accuracies are different for subjects of Data 1 and Data 2 with the increasing of the *K*. However, we note that the average test accuracies are higher when *K* is 16. Therefore, we choose *K* to be 16 in this study.

### Parameter analysis of improved novel global harmony search

The selection of HMS depends on the specific problem. The bigger HMS cause that the search space of the algorithm is larger, which is easier to find the global optimal solution. However, a larger HMS will inevitably bring about an increase in the running time of the algorithm. Therefore, considering the efficiency and diversity of the algorithm, an appropriate HMS value for the INGHS algorithm is very vital. Mutation in INGHS is an auxiliary search operation whose main purpose is to preserve the diversity of group. Generally, a small *P*_*m*_ value may lead to rapid convergence of the algorithm, which will easily generate local optimization solutions. However, a higher *P*_*m*_ value will make INGHS algorithm tend to be purely random search, resulting in slow convergence of the algorithm and greatly affecting the efficiency of problem solving. Therefore, an appropriate *P*_*m*_ value can not only prevent the algorithm from falling into local optimum and keep the diversity of solutions, but also make the algorithm timely converge. To select the optimal HMS and *P*_*m*_, this study analyzed the effects of different HMS and *P*_*m*_ on three data sets, and HMS values are set as 5, 10, 20, and 30. If the total number of variables 1 ≤ *x*_*n*_ ≤ 4, *P*_*m*_ is reasonably selected from the range region of [0.2 × (1 − 50%), 0.2 × (1 + 50%)]. Otherwise, *P*_*m*_ is selected from the region of [(1 − 50%) / x_*n*_, (1 + 50%) / x_*n*_] (Zou et al., 2010). The *P*_*m*_ value in this study is most reasonable between 0.1 and 0.3, so the *P*_*m*_ value is set to 0.1, 0.15, 0.2, 0.25, and 0.3. Set the number of channels to *K* = 16. The classifier uses LDA.

Table 2 shows the mean test accuracy of all subjects for Data 1 under different HMS and *P*_*m*_. It can be seen from the table that when HMS = 10 and *P*_*m*_ = 0.15, the average test accuracy is up to 78.43%. Therefore, for Data 1, the HMS and *P*_*m*_ are set to 10 and 0.15, respectively. Table 3 shows the mean test accuracy of all subjects for Data 2 under different HMS and *P*_*m*_. It can be seen that when HMS = 10 and *P*_*m*_ = 0.2, the average test accuracy is the highest, reaching 87.78%. Therefore, for Data 2, parameters HMS and *P*_*m*_ are selected as 10 and 0.2, respectively. Table 4 shows the mean test accuracy of all subjects for Data 3 under different HMS and *P*_*m*_ values. It can be seen that the average test accuracy is up to 81.76%. Therefore, for Data 3, HMS and *P*_*m*_ were selected as 20 and 0.25, respectively.

**TABLE 2:** Mean test accuracy (%) of HMS and *P*_*m*_ changes of INGHS of all subjects for Data 1.

*P*_*m*_	HMS			
	5	10	20	30
0.1	76.57	75.71	77.43	76.50
0.15	75.07	**78.43**	76.64	76.07
0.2	72.64	74.07	75.86	75.00
0.25	74.07	73.14	74.93	73.07
0.3	74.36	74.93	73.71	76.14

The bold value indicates the highest average accuracy.

**TABLE 3:** Mean test accuracy (%) of HMS and *P*_*m*_ changes of INGHS of all subjects for Data 2.

*P*_*m*_	HMS			
	5	10	20	30
0.1	85.43	86.07	86.57	85.21
0.15	84.00	85.57	85.86	85.57
0.2	85.71	**87.78**	86.21	87.77
0.25	85.79	85.50	84.86	85.50
0.3	85.00	87.28	86.33	86.36

The bold value indicates the highest average accuracy.

**TABLE 4:** Test accuracy (%) of HMS and *P*_*m*_ changes of INGHS of all subjects for Data 3.

*P*_*m*_	HMS			
	5	10	20	30
0.1	72.69	77.87	78.79	74.35
0.15	77.41	78.34	79.71	76.02
0.2	77.78	74.91	78.24	79.63
0.25	78.98	81.67	**81.76**	75.47
0.3	76.57	79.91	78.24	80.00

The bold value indicates the highest average accuracy.

In this study, all experimental simulations are implemented by using MATLABR2019b on a Windows personal computer with Core i5-9500H 3.00 GHz CPU and RAM 8.00 GB. For fair comparisons, PSO (Xu et al., 2014), and the ABC (Miao et al., 2017a) with the recommended parameter setting were also employed to find the optimal frequency-temporal parameters. Moreover, the training data, test data, and the prepossessing methods are consistent. The parameter values of all the algorithms are listed in Table 5. Similarly, the number of iterations (Ni) for the all of algorithm is 100.

**TABLE 5:** The parameters of PSO, ABC, and INGHS.

Data sets	Parameters		
	
	INGHS	PSO	ABC
Data 1	HMS = 10, *P*_*m*_ = 0.15	Population size = 10, c1 = c2 = 2.0, *w* = 0.9−(0.4 / Ni) × u	Colony size = 10, number limit = 5
Data 2	HMS = 10, *P*_*m*_ = 0.2	Population size = 10, c1 = c2 = 2.0, *w* = 0.9−(0.4 / Ni) × u	Colony size = 10, number limit = 5
Data 3	HMS = 20, *P*_*m*_ = 0.25	Population size = 20, c1 = c2 = 2.0, *w* = 0.9−(0.4 / Ni) × u	Colony size = 20, number limit = 5

× u is the current iteration, Ni is the number of iterations, c1 and c2 are acceleration weight, and *w* is inertia weight.

## Results and discussion

### Channel selection

**FIGURE 6:**
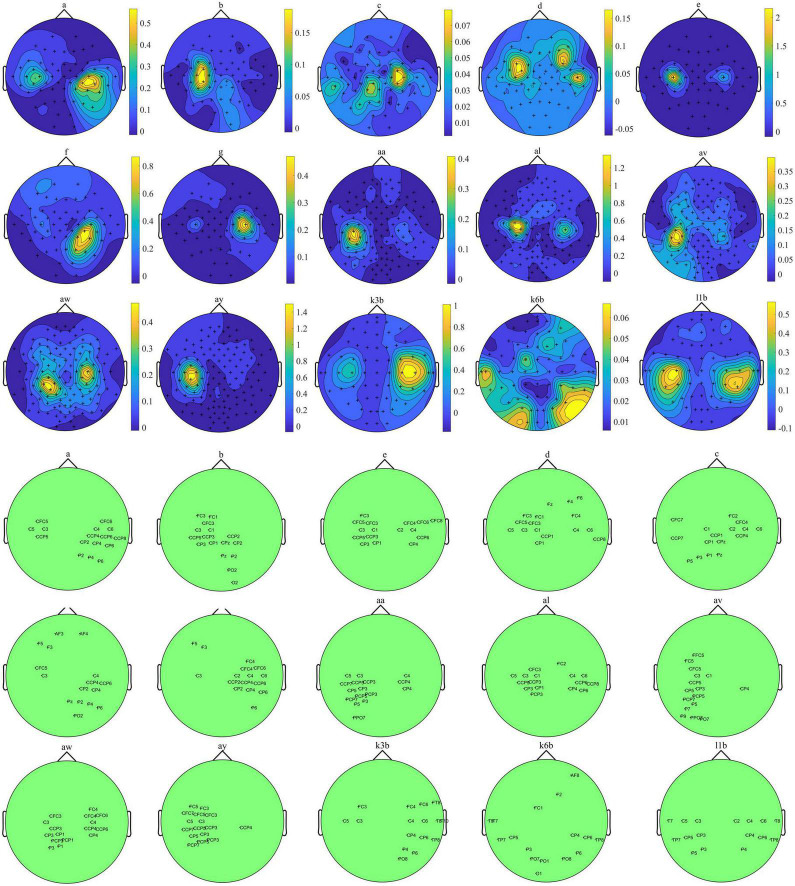
The topographic map of channels’ discriminative power distributions of each subject.

Figure 6 depicts channels’ discriminative power distributions and the selected channels for all subjects. The darker yellow indicates the greater the FDC value of this channel, on the contrary, the lighter blue indicates the smaller the FDC value of corresponding channel. As shown in Figure 6, for subjects of Data 2, the selected channel sets distributed in the left motor cortex area. Moreover, for subjects b, c, d, e, and g, the selected channels mainly located in right and left motor cortex area, which corresponds to MI of left hand and right hand. For subjects a and f, selected channels tend to locate in the right and central motor cortex area. It is consistent with the neurophysiological principle which is MI of the left hand and foot corresponding to the right and central motor cortex area. Furthermore, for subjects k3b to l1b, the channels correspond to higher FDC values are distributed in the adjacent area at C3 and C4 positions. However, the selected channels for each subject are distinguishing each other due to individual differences.

### Test classification comparison

Aiming to evaluate the effectiveness of the proposed INGHS method for optimizing frequency-time selection, we compared the test classification between the proposed INGHS method and ABC, PSO, and the traditional CSP method in Table 6. For PSO, ABC, and INGHS feature optimization methods, the number of channels *K* is 16. Furthermore, the CSP method with 16-channels employed the fixed time windows (0–4 s for data set 1 and IIIa, and 0–3.5 s for data set IVa) and the frequency band selected 5–40 Hz. Note that all the methods in Table 6 are evaluated by standard competition procedure. Each value in Table 6 is the 10-fold cross-validation mean test accuracy. Table 6 shows that for LDA and SVM classifiers, the average accuracy rate improvement achieved by INGHS was 12.9 and 11.6% in comparison with the classical CSP method. Thus, the proposed INGHS method achieved higher average classification accuracies compared to the traditional CSP method. In addition, the average accuracy rate improvements achieved by INGHS based on LDA were 1.6 and 4.1% in comparison with the ABC and PSO methods. Meanwhile, for the SVM classifier, the average classification accuracy obtained by the proposed INGHS method is higher than 0.1% (ABC) and 3.9% (PSO). The average test accuracy for INGHS is found to be slightly higher as compared to the accuracy achieved by ABC and PSO based on two classifiers. Furthermore, the Wilcoxon signed-rank test was used to analyze the statistical differences between the CSP method and the proposed INGHS method. The classification result of INGHS was significantly better than that of the CSP method (*p* < 0.001).

**TABLE 6:** Test classification accuracy (%) of different methods applied on datasets 1–3.

Participants	CSP		PSO		ABC		INGHS	
				
	LDA	SVM	LDA	SVM	LDA	SVM	LDA	SVM
a	60.0	62.0	66.0	74.0	66.0	70.0	74.0	71.3
b	61.5	61.5	58.5	65.5	57.0	68.0	69.0	62.5
c	71.5	72.0	68.5	71.5	68.0	78.5	80.5	80.0
d	68.0	66.0	79.0	86.0	85.0	87.5	82.0	81.0
e	81.0	80.0	91.0	88.5	93.5	91.5	91.5	90.5
f	71.0	71.5	63.0	75.0	80.5	75.5	71.5	80.0
g	60.5	64.5	76.5	88.5	83.5	83.5	80.5	83.5
aa	67.1	66.4	84.3	70.0	87.1	73.2	81.8	75.0
al	79.3	73.6	97.5	98.2	97.5	90.5	96.1	92.0
av	57.1	62.9	71.4	55.4	64.6	66.4	71.8	69.3
aw	71.8	81.4	91.8	78.6	93.2	84.3	93.9	90.0
ay	75.4	62.9	92.9	77.1	92.9	91.5	95.4	94.3
k3b	80.0	77.2	90.0	84.4	92.6	93.2	92.8	91.1
k6b	60.8	62.5	53.3	55.0	63.3	65.5	60.8	60.5
l1b	74.2	71.7	86.7	84.17	84.0	89.2	91.7	90.2
Mean ± SD	69.3 ± 7.9	69.1 ± 6.8	78.1 ± 13.9	76.8 ± 12.2	80.6 ± 13.3	80.6 ± 10.1	82.2 ± 11.1	80.7 ± 10.9

For the swarm intelligence optimization algorithm, PSO adjusts all variables of each solution during each iteration and the ABC algorithm adjusts one variable of each solution, whereas the INGHS algorithm adjusts each variable independently based on all of the existing vectors. This feature could increase the flexibility of the algorithm and produce better solutions. In addition, the steps and the structure of the INGHS algorithm are relatively simple. In summary, on the one hand, the proposed INGHS method could effectively optimize the time and frequency parameters and achieve better test accuracy compared with the CSP method. On the other hand, the test accuracy that is obtained by the time-frequency parameters selected by the proposed INGHS algorithm is slightly better than that obtained by ABC and PSO.

### Frequency-time analysis

**FIGURE 7:**
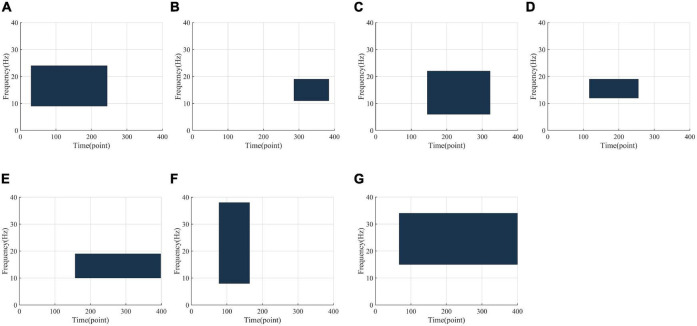
The optimal frequency-time region found by the INGHS approach based on LDA for subject **(A–G)**.

The optimal frequency-time zone optimized by the INGHS method based on LDA for Data 1 is shown in Figure 7. The figure shows the optimal frequency-time parameters corresponding to the highest test accuracy in the 10-fold cross-validation for each subject. It should be noted that, for all subjects, the optimal frequency band covers the μ (8–12 Hz) and β rhythms (13–30 Hz). However, they still vary a lot. Furthermore, we observe that the starting time are different in a small range for all the subjects and the optimal time lengths are different. Meanwhile, the optimal time segment of most subjects starts at 0.5 s after the visual cue which is consistent with the starting time point used in most literature (Thomas et al., 2009).

**FIGURE 8:**
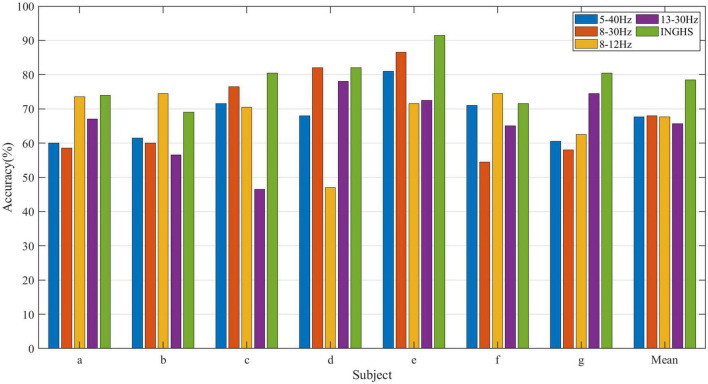
Comparison of classification accuracies obtained by INGHS and common frequency settings for Data 1.

For brain signal analysis, some common frequency bands including 8–30 Hz, μ (8–12 Hz), and β (13–30 Hz) have been popularly used in various EEG studies. Here, we also investigate the classification performance between the commonly used frequency band configuration and the frequency band and time configuration optimized by the INGHS method. The time segment was 0–4 s, and the classifier was LDA. It should be emphasized that all the comparisons were made when the number of channels *K* was 16. Figure 8 presents the comparison of classification accuracies obtained by standard competition procedure with INGHS and common frequency settings for Data 1. The results show that the optimal frequency-time parameter based on INGHS achieved a better average test accuracy in comparison with the common frequency band setting. Specifically, the average test accuracy of INGHS is 78.43%, which is higher than 10.79, 10.43, 10.72, and 12.72% of the CSP method with 5–40, 8–30, 8–12, and 13–30 Hz, respectively. Therefore, it is suggested that INGHS could adaptively select the optimal time-frequency parameters and achieve the better classification performance.

### Comparison of spatial patterns

**FIGURE 9:**
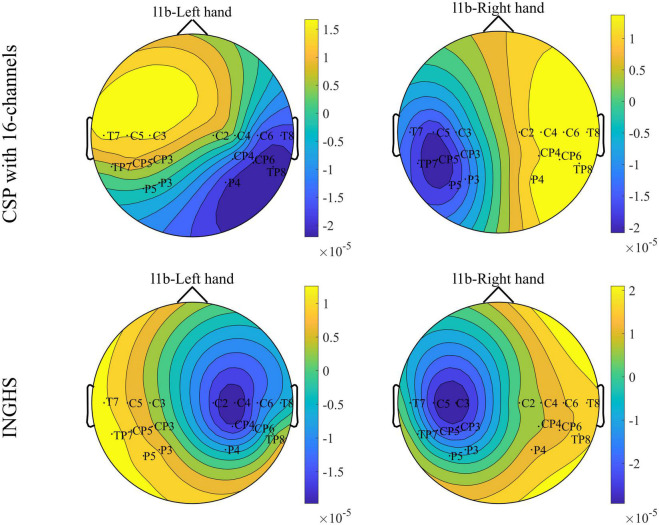
Comparison of spatial patterns for the subject “l1b.”

To better interpret the experimental results, we visualized the spatial pattern derived by the traditional CSP method and the INGHS-based method. A pair of spatial patterns is composed of the first and last columns of *W*^−1^ (*W* is the spatial filter as in section “Feature extraction and classification”). For the traditional CSP method, the fixed frequency band (5–40 Hz) and time segment (0–4 s) are applied to the training data to obtain the spatial filter *W*. Meanwhile, the based-INGHS method applies the optimized frequency band and time period to the training data to obtain the spatial filter *W*. The channel used by INGHS and CSP method is the optimal 16-channel mode after channel selection approach. It should be noted that the training data used by CSP and INGHS are consistent. In Figure 9, a comparison of spatial patterns between the traditional CSP method with 16-channels and the INGHS-based method for “l1b” are displayed. The results indicated that compared with the traditional CSP method, the spatial pattern based on the INGHS method had significant ERD, which is concentrated around C3 and C4. When the unilateral MI, there was significant ERD in SMR at the contralateral hemisphere (Pfurtscheller et al., 2006). Moreover, the information features of sensorimotor areas are closely related to ERD, which provides important discriminant information for the decoding of motor imagination tasks (Blankertz et al., 2007). The obvious ERD in SMR derived by the INGHS-based method leads to better decoding accuracy in Table 6. Additionally, these analyses provide explicit evidence for the superior decoding performance of our proposed INGHS method over the traditional CSP method.

### Computational cost comparison

The computational time of the different methods for three data sets is shown in Table 7. The running time mainly refers to the iteration time of the algorithm, excluding preprocessing and channel selection. The computational time denotes the average running time of all subjects in a single data set. As shown in Table 7, compared with PSO and ABC, the average time spent by the LDA-based INGHS method for all of the data was reduced by 78.2 and 85.2%, respectively. The results show that the proposed method in our study takes less time than PSO and ABC. The main reason is that in contrast to INGHS algorithms in which a unique solution is generated at each iteration, population-based meta-heuristic algorithms (PSO and ABC) take more time to maintain a set of solutions that evolve at each iteration. Although our proposed INGHS method takes less time than other methods, we expect to dramatically reduce the computational cost to speed up the training phase.

**TABLE 7:** Computational time (s) comparisons of different methods for three data sets.

Datasets	PSO	ABC	INGHS
Data 1	1,064.4	1,974.5	245.9
Data 2	1,410.7	1,393.2	311.4
Data 3	890.8	1,575.5	177.5
Mean	1,121.9	1,647.7	244.9

### Comparison of the existed methods

Table 8 shows the test classification accuracy between the proposed INGHS method and the existed methods (FBCSP and SFBCSP). The time length was 0–3.5 s for Data 2 on the FBCSP and SFBCSP. For the FBCSP, the sub-frequency bands are divided into 4–8, 6–10, 8–12…, 36–40 Hz. CSP features are extracted for the whole time window in each sub-frequency band, and then the Mutual Information based Best Individual Feature (MIBIF) selection algorithm is used. CSP features of the frequency band are automatically selected. Based on the mutual information value of a single feature, the features corresponding to the first four sub-frequency bands are selected for subsequent training and testing. For SFBCSP method, sub-band division are the same as FBCSP, the LASSO is used for feature optimization. The 16-channel mode for the FBCSP and SFBCSP is the same as INGHS. Each value in Table 8 is the fivefold cross-validation mean test accuracy. The LDA is used as classification method. The proposed INGHS method achieved higher average classification accuracies compared to the FBCSP and SFBCSP methods. In addition, the average accuracy rate improvements achieved by INGHS were 3.5 and 2.5% in comparison with the FBCSP and SFBCSP methods.

**TABLE 8:** Test classification accuracy (%) of the INGHS and existed methods.

Participants	FBCSP	SFBCSP	INGHS
aa	76.8	77.9	82.9
al	94.3	96.1	96.4
av	67.5	66.1	67.9
aw	89.3	90.7	92.1
ay	86.8	88.6	92.5
Mean ± SD	82.9 ± 10.7	83.9 ± 11.9	86.4 ± 11.5

### Limitations and extensions

The proposed frequency-temporal parameters optimization method based on INGHS could yield better test accuracy compared with the traditional CSP method. Furthermore, compared with PSO and ABC, the proposed method takes less computation time because of the simple iterative principle of the INGHS algorithm which can quickly converge to the global optimal value. However, the channel selection step precedes time-frequency optimization, and we will further explore the impact of spatial-frequency-time domain simultaneous optimization on classification performance in the future. Moreover, the proposed INGHS method only validates the binary classification performance of MI-BCI systems, and further research is needed to apply the proposed time-frequency parameter optimization algorithm to multi-classification problems.

## Conclusion

In this study, an approach of frequency-time feature optimization based on the INGHS is proposed for MI EEG decoding. Three EEG datasets are used to verify the effectiveness of proposed INGHS method. The proposed method could improve classification accuracy in comparison to the classical CSP method. Moreover, the average test accuracy achieved by the INGHS is slightly better than that obtained by ABC and PSO based on LDA and SVM. Furthermore, the INGHS algorithm is superior to PSO and ABC in running time. The results demonstrate that the optimal frequency band and time interval provided by the INGHS algorithm could indeed improve the classification accuracy. Future studies will investigate the performance of our proposed INGHS method on other types of BCI systems.

## Data availability statement

The original contributions presented in this study are included in the article/supplementary material, further inquiries can be directed to the corresponding authors.

## Author contributions

BS performed the study, analyzed the data, and was responsible for drafting the manuscript. XC, ZY, and SY helped to perform the study and revised the manuscript. FZ, BW, and JW analyzed and discussed the results. All authors read and approved the final manuscript.
